# Poly(lactide-*co*-trimethylene carbonate) and Polylactide/Polytrimethylene Carbonate Blown Films

**DOI:** 10.3390/ijms15022608

**Published:** 2014-02-14

**Authors:** Hongli Li, Jiangping Chang, Yuyue Qin, Yan Wu, Minglong Yuan, Yingjie Zhang

**Affiliations:** 1 Engineering Research Center of Biopolymer Functional Materials of Yunnan, Yunnan University of Nationalities, 134, Yi Er Yi Avenue, Kunming 650031, Yunnan, China; E-Mails: hongli.li1982@gmail.com (H.L.); jiangpingc0828@gmail.com (J.C.); 2 Institute of Chemical Engineering, Kunming University of Science and Technology, Kunming 650550, Yunnan, China; E-Mails: rabbqy@163.com (Y.Q.); 5yan.zi@163.com (Y.W.)

**Keywords:** polylactide, polytrimethylene carbonate, poly(lactide-*co*-trimethylene carbonate), polylactide-polytrimethylene carbonate, film blowing

## Abstract

In this work, poly(lactide-*co*-trimethylene carbonate) and polylactide/polytrimethylene carbonate films are prepared using a film blowing method. The process parameters, including temperature and screw speed, are studied, and the structures and properties of the P(LA-TMC) and PLA/PTMC films are investigated. The scanning electron microscope (SEM) images show that upon improving the content of TMC and PTMC, the lamellar structures of the films are obviously changed. With increasing TMC monomer or PTMC contents, the elongation at the break is improved, and the maximum is up to 525%. The water vapor permeability (WVP) results demonstrate that the WVP of the PLA/PTMC film increased with the increase in the PTMC content, whereas the WVP of the P(LA-TMC) film decreased. Thermogravimetric (TG) measurements reveal that the decomposition temperatures of the P(LA-TMC) and PLA/PTMC films decrease with increases in the TMC and PTMC contents, respectively, but the processing temperature is significantly lower than the initial decomposition temperature. P(LA-TMC) or PLA/PTMC film can extend the shelf life of apples, for instance, like commercial LDPE film used in fruit packaging in supermarkets.

## 1. Introduction

Recently, environmental concerns and disruptions of oil resources have led to increased efforts in the use of biodegradable polymers at an industrial scale, especially in packaging. Blown films are widely used to produce a variety of packaging films and bags [1]. In the current market, these films are prepared with polyethylene (PE), low-density polyethylene (LDPE), high-density polyethylene (HDPE) and polypropylene (PP). These polymers are obtained from petroleum and are non-biodegradable, and the environment could be polluted with the waste film products [2]. Therefore, biodegradable packing materials have great market prospects. The use of biodegradable polyesters (e.g., polylactide, polycaprolactone and poly(trimethylene carbonate)) in temporary biomedical applications has increased significantly over the past decade [3–5]. Polylactide is the most promising material as a substitute for plastic films by film blowing.

Polylactide (PLA) is a well-known polymer that has been studied extensively for various biomedical applications due to its acceptable biocompatibility and inherent biodegradability [6–8]. Moreover, because of its thermoplasticity, high modulus, high strength and biodegradability, the polymer is widely studied as a packing material [9,10]. As a result of its strong rigidity, pure PLA is not suitable for preparing films by blow molding. Poly(trimethylene carbonate) (PTMC) is an interesting candidate to introduce modifications to rigid PLA [11–13]. PTMC of aliphatic carbonate is also biodegradable by lipase, and it is synthesized by a ring-opening polymerization of trimethylene carbonate (TMC). At room temperature, it is a rubbery and flexible material [14,15]. In the present study, the PLA/PTMC blending films and poly(lactide-*co*-trimethylene carbonate) films are prepared using hot pressing or solvent methods, and these materials are mainly used in biomedical areas, for example, for bioabsorbable sutures, implantable medical devices, tissue engineering scaffolds and controlled drug delivery systems [16–20].

In this work, we have investigated the structures and properties of poly(lactide-*co*-trimethylene carbonate) and polylactide/polytrimethylene carbonate films prepared by blow molding. The poly(lactide-*co*-trimethylene carbonate) copolymers are prepared by ring opening polymerization. The chemical structure and molecular weight of the P(LA-TMC) polymers are determined with NMR and GPC. The aim of this study is to analyze the crucial parameters of the preparation of films. In addition, the structures of the films are explored using Fourier transform infrared spectroscopy (FTIR) and scanning electron microscopy (SEM). The mechanical properties, water vapor permeability and thermal stability of the composite films have also been examined.

## 2. Results and Discussion

### 2.1. The Characteristics of the PTMC and P(LA-TMC) Polymers

To facilitate understanding, the P(LA-TMC) polymers with the lactide ratios of 50%, 70%, and 90% are recorded as P(LA-TMC)-5, P(LA-TMC)-7, and P(LA-TMC)-9, and the blended composites of polylactide and polytrimethylene carbonate with polylactide ratios of 50%, 70%, and 90% are recorded as PLA/PTMC-5, PLA/PTMC-7, and PLA/PTMC-9. The molecular weights and molecular distributions of the PTMC and Poly(lactide-*co*-trimethylene carbonate) polymers with different ratios were determined by GPC. The basic characteristics of the PTMC and P(LA-TMC) polymers are presented in Table 1.

**Figure 1.**
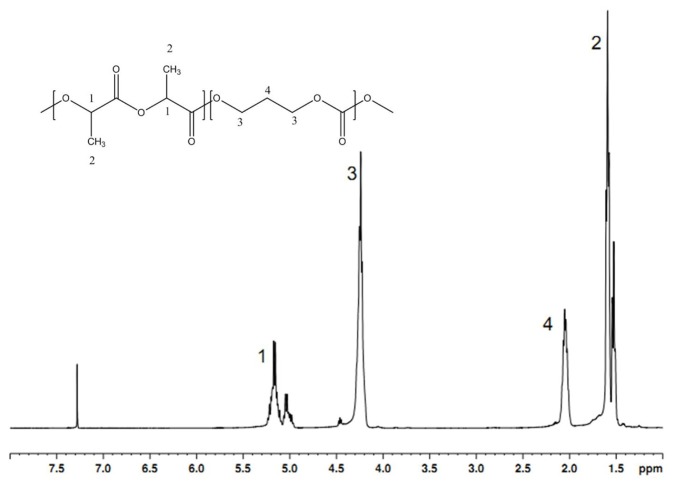
The ^1^H-NMR spectrum of the P(LA-TMC) polymer.

The structures of the P(LA-TMC) polymers are determined by the ^1^H-NMR spectrum. As shown in Figure 1, signals in the 4.9–5.2 ppm zone and at 1.5 ppm are assigned to –CH– and –CH_3_– of the lactyl units, and those at 4.2 and 2.0 ppm are assigned to the –CH_2_– of the TMC units. Signals in the 5.0–5.2 ppm zone can be divided in two groups: the downfield group is approximately 5.2 ppm and belongs to the main chain lactyl units, and the upfield group is approximately 5.0 ppm and is assigned to the lactyl units linking to TMC units. This result indicates that P(LA-TMC) copolymers were successfully obtained.

### 2.2. The Processing Parameters of Film Blowing

**Figure 2.**
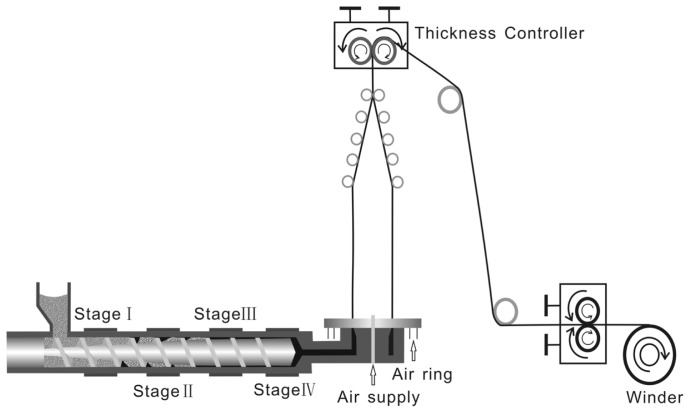
The processing flow diagram of the extrusion blown film.

The poly(lactide-*co*-trimethylene carbonate) and polylactide/polytrimethylene carbonate films are prepared by blow molding, and Figure 2 is the processing flow diagram of the extrusion blown film. The effects of the temperature and screw speed of the film blowing are shown in Tables 2 and 3. The optimum processing parameters can been obtained from Tables 2 and 3. When the processing temperature of each section is 110, 150, 150, or 137 °C and the screw speed is 40 pr/min, smooth P(LA-TMC) films are prepared. Meanwhile, smooth PLA/PTMC films are prepared when the processing temperature of each section is 170, 185, 185, or 175 °C at a 40 pr/min screw speed. Comparing ^Table 2 to Table 3^, we can see that the processing temperature is different between the P(LA-TMC) film and the PLA/PTMC film. The reason could be that the binding mode and the microstructures of the two films are different. Using our best preparation process, the P(LA-TMC) films are completely transparent, but the PLA/PTMC film is translucent. The macroscopic features of the PLA/PTMC film are similar to Adamus’s report [21]. The P(LA-TMC) film prepared by our method is soft and completely transparent, and this is different from existing literature work in the report.

### 2.3. FTIR Measurements

**Figure 3.**
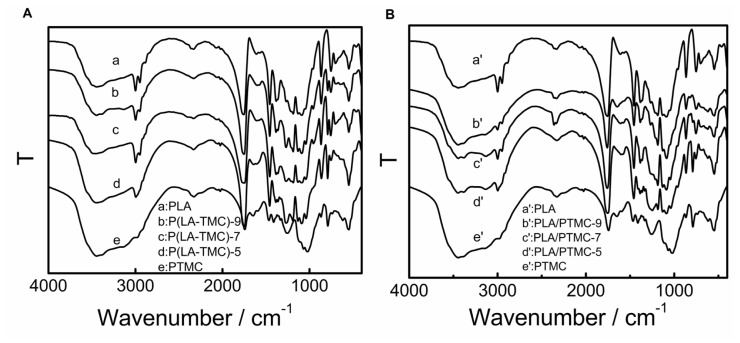
FTIR spectra for PLA, PTMC, P(LA-TMC) (**A**) and PLA/PTMC (**B**).

FTIR spectra are recorded for all samples to investigate the chemical structure. Figure 3 shows the FTIR spectra of PLA, P(LA-TMC) and PLA/PTMC. Comparing the P(LA-TMC) polymers to the PLA/PTMC blends, and they are similar to the curve of PLA. As shown in Figure 3, the IR bands at 2997 and 2946 cm^−1^ are assigned to the CH stretching region, ν_as_CH_3_ and ν_s_CH_3_ modes. The C–O stretching region is observed as a band at 1747 cm^−1^. The region between 1500 and 1360 cm^−1^ is characterized by the 1452 cm^−1^ CH_3_ band. The 1380 and 1360 cm^−1^ peaks could be ascribed to the CH deformation and asymmetric bands. In the region of 1000 to 1300 cm^−1^, it is possible to observe the C–O stretching modes of the ν_O–C_ asymmetric mode at 1180 cm^−1^ and the ester groups at 1267 cm^−1^. Two bands related to the crystalline and amorphous phases of PLA were found at 868 and 756 cm^−1^. The peak at 868 cm^−1^ can be assigned to the amorphous phase and the peak at 756 cm^−1^ to the crystalline phase. Similar theory has been reported in the literature [22,23]. However, we find that with the increase of TMC or PTMC, the band at 2946 cm^−1^ decreases. This could be due to the ν_s_CH_3_ being affected by steric hindrance and hydrogen bonding.

### 2.4. SEM Measurements

**Figure 4.**
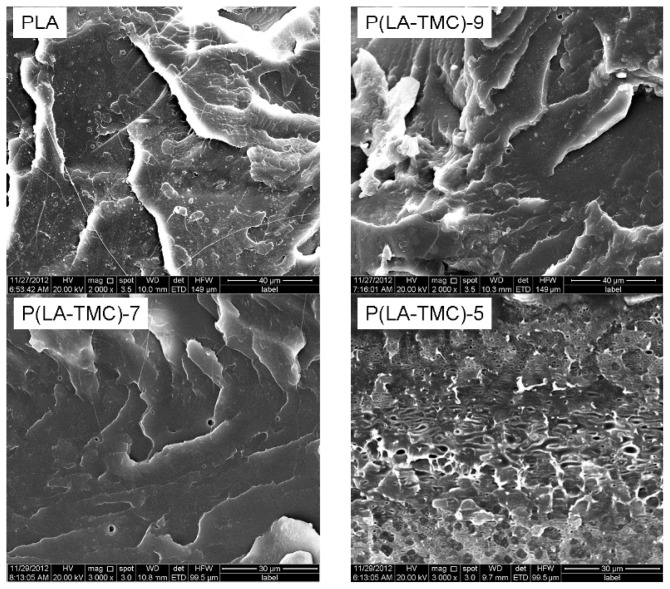
SEM images of PLA and P(LA-TMC) films with different TMC contents.

**Figure 5.**
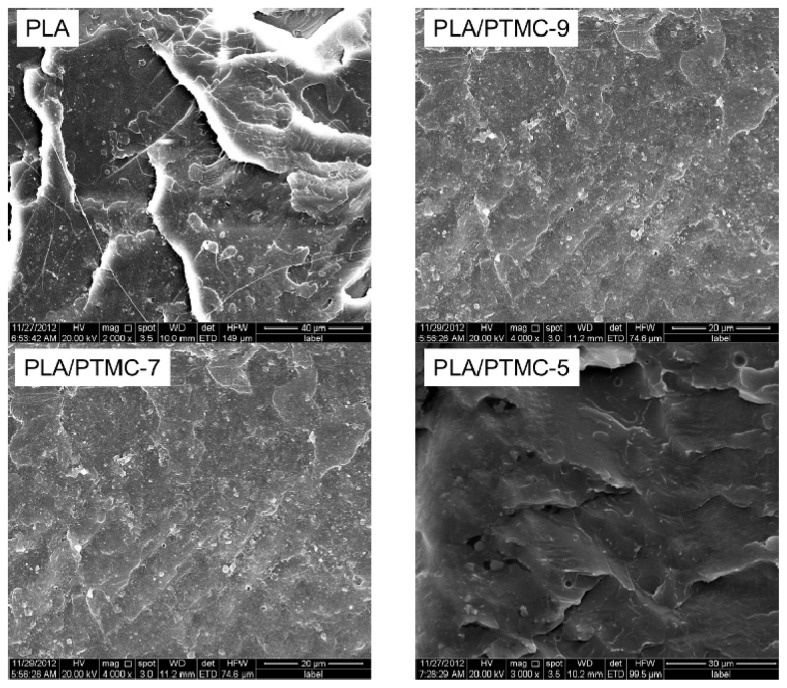
SEM images of the PLA and PLA/PTMC films with different PTMC contents.

Figures 4 and 5 show SEM micrographs of the cross sections of pure PLA, P(LA-TMC) and PLA/PTMC films. From Figures 4 and 5, we can see that the cross section of the PLA film is a lamellar structure, and by improving the content of TMC and PTMC, the morphology structure is changed. As shown in Figure 4, the lamellar structure gradually disappears with increasing contents of TMC monomer and when the TMC monomer is increased to 50%, porous morphology appears in the cross section of the P(LA-TMC) films. Figure 5 shows the relation of the filling effect of PTMC to that of the PLA/PTMC films, which look smoother than the P(LA-TMC) films. Nevertheless, when the PTMC content is 50%, there is a significant difference between PLA/PTMC-5 and P(LA-TMC)-5. The differences of the physical and chemical properties are induced by microstructure changes, and the changes of the mechanical properties and WVP performance will better illustrate this point in the following study.

### 2.5. Mechanical Properties Analysis

**Figure 6.**
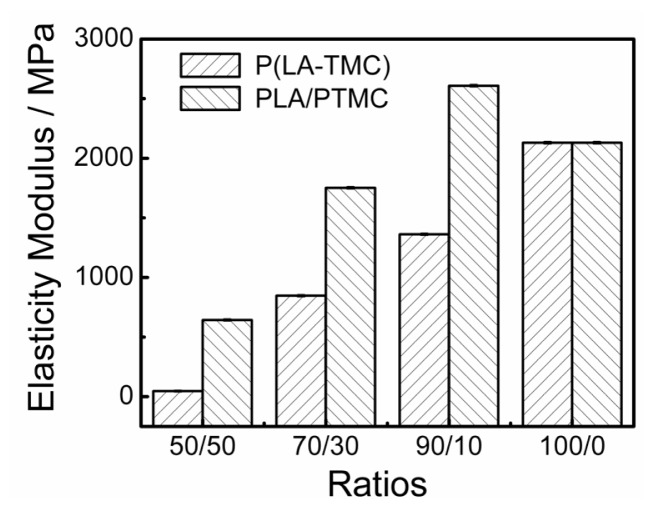
The elasticity modulus for P(LA-TMC) and PLA/PTMC films with different ratios.

**Figure 7.**
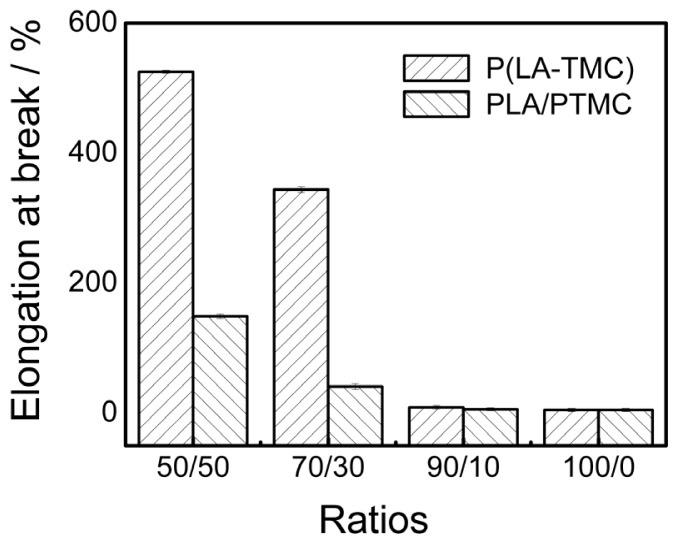
The elongation at break for P(LA-TMC) and PLA/PTMC films with different ratios.

The mechanical properties of the obtained films are also dependent on these different structures. Figures 6 and 7 show the elasticity modulus and elongation at break of the P(LA-TMC) and PLA/PTMC films. As shown in Figures 6 and 7, both for the P(LA-TMC) films and the PLA/PTMC films, the elasticity modulus is gradually decreased with the increase of TMC monomer or PTMC, but the elongation at break is improved. The maximum of the elongation at break is up to 525%. The increase of the elongation at break shows an improvement of the toughness, thus the blown film application can enlarge. The reason for this phenomenon is that the microstructure of the composite-based PLA is changed. As shown in Figures 4 and 5, the smoothness of the blown film is better with increased TMC and PTMC. The maximum is much higher than the elongation at break of the PLA and PLA/PTMC films in the current literatures [9,17]. The increased elongation at break is due to the improved smoothness of the blown film, but the improved smoothness also leads to a reduction in rigidity, so that a lower elasticity modulus is achieved.

### 2.6. WVP Measurement

**Figure 8.**
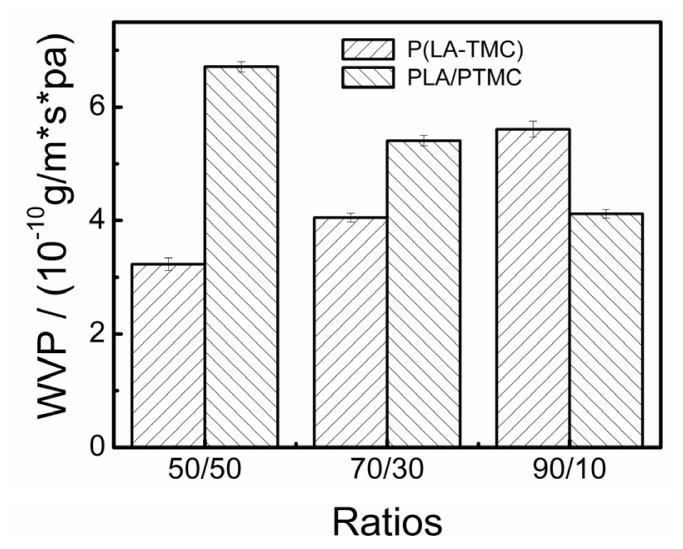
WVP of the P(LA-TMC) and PLA/PTMC films with different ratios.

A main function of food packaging material was to impede gas and water vapor transfer between food and the surrounding atmosphere, so WVP of the films should be as low as possible [24]. Figure 8 shows the water vapor permeability (WVP) of the composite films. The property relates to the network structure and the available hydrophilic groups on the channel surface. From Figure 8, we can see that the P(LA-TMC) films and the PLA/PTMC films have an opposite trend and that the water vapor permeability of the films is better than the PLA film reported in the literature [25,26]. The WVP of PLA/PTMC film increased with the increase of PTMC content, but the WVP of P(LA-TMC) film declined. It may be due to the changes of morphological structure. When the TMC content reaches 50%, there are many intensive small pores in the cross section of P(LA-TMC) film-5. Because the gap between layers is more conducive for water vapor to pass through than small pores, the barrier property of P(LA-TMC)-5 film is the best in all samples.

### 2.7. TG Measurements

**Figure 9.**
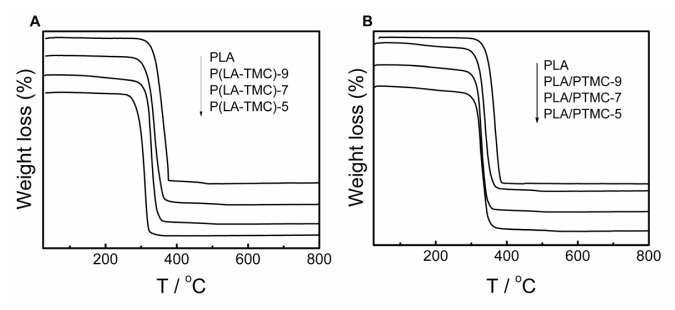
TG curves of PLA, P(LA-TMC) films (**A**) and PLA/PTMC films (**B**).

Concerning the TG and DTG, experiments are conducted in the scanning mode in a flowing nitrogen atmosphere for each film, and the heating rate is 10 °C/min. Figure 9 shows the TG curves of the PLA, P(LA-TMC) and PLA/PTMC films. In the scanned temperature range (35–800 °C), all films degraded through a single stage without the formation of appreciable residue. Because the thermal stability of polymers is connected with both the initial decomposition temperature and the temperature at the maximum rate of weight loss, the data of the initial decomposition temperatures, as well as the decomposition temperatures, of our composite films are considered and reported in Table 4. As shown in Figure 9 and Table 4, the initial decomposition temperatures and decomposition temperatures of the composite films are lower than the pure PLA film. The decomposition temperatures of the P(LA-TMC) and PLA/PTMC films decrease with an increase of TMC and PTMC, respectively, because the decomposition temperature of PLA is higher than PTMC. Furthermore, the initial decomposition stage is mainly considered the degradation of C–C skeleton in TMC chain. The decomposition stage is considered to be the degradation of polylactide chain. In addition, in Figure 9B, the initial decomposition temperatures of the PLA/PTMC films are similar to the decomposition temperatures of PTMC reported in literature [23,27]. Table 4 shows that when the TMC and PTMC contents are greater than 70%, the decomposition temperatures of the films are equal. However, when the content is 50%, the decomposition temperatures are different, due to microstructure differences. The results further support the previous SEM and elongation at break measurements.

### 2.8. Effects of P(LA-TMC) Film and PLA/PTMC Film on the Shelf Life of Apples

Weight loss, firmness, acidity, and sensory scores of apples were listed in Table 5. There was no significant (*p* > 0.05) difference among P(LA-TMC)-7, PLA/PTMC-7, and LDPE samples, with respect to weight loss, firmness, acidity, and sensory scores. Control apples spoiled in less than two weeks, whereas apples packaged in P(LA-TMC)-7, PLA/PTMC-7, or LDPE film displayed a shelf life of three weeks. Therefore, P(LA-TMC)-7 or PLA/PTMC-7 film can be used as food packaging material like commercial LDPE film.

## 3. Experimental Section

### 3.1. Materials

Lactic acid (LA) and polylactide (PLA) were purchased from Nature Works Co. Ltd. (Blair, NE, USA), and PLA had a density of 1.24 g/cm^3^ and a weight-average molecular weight (*M*_w_) of 2.50 × 10^5^ g/mol. PTMC is not available commercially; therefore, the polymer was synthesized in our laboratory. The method for PTMC polymerization was based on that described in the literature [21]. The polymerization of the trimethylene carbonate monomer was performed by melting with a stannous octoate catalyst under a nitrogen atmosphere. The stannous octoate was added to the monomer at 0.15% (g/g), and the polymerization was conducted for approximately 5 h at 120 °C. The resulting polymer was purified by dissolving with dichloromethane, precipitating with methanol and drying under reduced pressure at 60 °C.

### 3.2. Polymerization of Poly(lactide-co-trimethylene carbonate)

The molten ring opening polymerization of different weight ratio (50%, 70%, 90%) lactide and trimethylene carbonate (the total monomer is 400 g) was performed in a 500 mL single-neck flask equipped with a magnetic stirrer, using 1.5‰ (g/g) SnOct as catalyst. The reactants were filled with nitrogen and degassed in vacuum three times then sealed. The reactants were immersed into an oil bath at 100 °C until melted and the oil bath was increased to 130 °C, kept under magnetic stirring. After 5 h reaction, the polymer was removed from the flask by dissolution in chloroform and was purified by precipitation in ethanol. The residue was dried under vacuum at 60 °C by P_2_O_5_.

### 3.3. Preparation of Blown Films

The poly(lactide-*co*-trimethylene carbonate) films and polylactide/polytrimethylene carbonate films with the polylactide ratios of 50%, 70%, 90% were prepared using blow-film extrusion with an extruder (LSJ 20, Shanghai, China). The die diameter is 30 mm and its gap thickness is 100 μm. The blow up ratio is two times and the pulling speed is 357.2 mm/min. The films were blow molded with a predetermined screw speed at a designated temperature. With these settings, the film average thickness is approximately 20 μm (0.02 mm).

### 3.4. ^1^H-NMR Measurement

The ^1^H spectra of the copolymers were recorded with an NMR (Bruker Avance III 400 MHz). Deuterated chloroform (CDCl_3_) was used as the solvent, and the chemical shifts were given with respect to tetramethylsilane (TMS). ^1^H spectra were obtained by 16 scans.

### 3.5. GPC Measurements

The molecular weights and molecular distributions of PTMC and P(LA-TMC) with different ratios were determined by GPC with a Waters Associates model ALC/GPC 244 apparatus at 40 °C with a differential refractometer as the detector, THF as the solvent, and calibration with polystyrene standards. Three specimens were tested under each condition.

### 3.6. FTIR Spectra Measurements

Fourier transform infrared spectroscopy (FTIR) spectra were obtained from the films equilibrated in a vacuum drying cabinet for 24 h at 60 °C by an FTIR spectrometer (Nicolet IS10, Franklin, MA, USA). All spectra were obtained with a resolution of 2 cm^−1^ in the range of 400–4000 cm^−1^. The spectra plots represent the average of 10 scans.

### 3.7. SEM Measurements

The cross-section morphology of the composite films was observed directly by a scanning electron microscope (Quanta200, FEI, Hillsboro, OR, USA) without sputter coating with conducting matter. The film sample was first frozen in liquid nitrogen and then lyophilized at −47 °C.

### 3.8. Mechanical Properties

The tensile testing was measured by a Universal Testing Machine (GMT-400, Shanghai, China). All samples were 105 mm × 17 mm cut from the blown-film samples. The tense speed was 50 mm/min. All reported results are the averages of at least three test specimens. The elasticity modulus and elongation at break of samples can be calculated by

(1) $$\sigma =\frac{p}{bd}$$

(2) $$E_{t}=\frac{\sigma }{ɛ}$$

(3) $$E_{b}=\frac{L-L_{0}}{L_{0}}$$

where *E_t_* is the elasticity modulus and *E_b_* is the elongation at break, σ is the tensile stress, *p* is the yield load, *b* is the width of sample, *d* is the thickness of sample, ɛ is strain, *L* is the original line distance of sample and *L*_0_ is the distance between the lines when the sample is broken.

### 3.9. Water Vapor Permeability (WVP) Measurement

The copolymer films and blended films were sealed on a top glass permeating cup containing distilled water (100% RH; 2337 Pa vapor pressure at 20 °C, which was placed in a desiccator at 20 °C and 0% RH containing silica (0 Pa vapor pressure). The cup was weighed at 1 h intervals over a period of 10 h. The WVP of the film was calculated by the following Equation [28]:

(4) $$WVP=w\times \chi \times A^{-1}\times t^{-1}\times {\left(P_{2}-P_{1}\right)}^{-1}$$

where *w* is the weight gain (g); χ is the film thickness (m); *A* is the area of exposed film (m^2^); *t* is time of gain (s); and (*P*_2_ − *P*_1_) is the vapor pressure differential across the film (Pa). This entire procedure was repeated thrice for each film type.

### 3.10. TG Measurements

Thermal weight loss of the PLA composite films was determined by a thermogravimetric analyzer (Netzsch TG 209F1, Selb, Germany) from 35 to 800 °C at a heating rate of 10 °C/min under nitrogen atmosphere. For each sample, approximately 4 mg specimen was taken for the analysis, and the flow rate of the nitrogen was 35 cm^3^/min.

### 3.11. Effects of P(LA-TMC) Film and PLA/PTMC Film on the Shelf Life of Apples

Apples were harvested and packaged in P(LA-TMC)-7 film, PLA/PTMC-7 film, and low density polyethylene (LDPE) film. Apples without packaging material were used as control group. The apples were then stored at room temperature for 28 days. Weight loss, firmness, acidity, and sensory evaluation were evaluated.

## 4. Conclusions

Polylactide and poly(trimethylene carbonate) are biodegradable materials that can be synthesized by ring-opening polymerization. Studies of the structure and properties of poly(lactide-*co*-trimethylene carbonate) and polylactide/polytrimethylene carbonate films prepared by blow molding can lead to the following conclusions. When the processing temperature of each section is 110, 150, 150, and 137 °C and the screw speed is 40 pr/min, smooth P(LA-TMC) films are prepared. Meanwhile, smooth PLA/PTMC films are prepared when the processing temperature of each section is 170, 185, 185, and 175 °C at the same screw speed. The mechanical properties, water vapor permeability and thermal stability of the composite films are related to the film microstructure and composite ratios. When the TMC and PTMC contents are 50%, the impact on the film properties is the greatest. For the P(LA-TMC) films and PLA/PTMC film, the performances have advantages and disadvantages. According to the requirements, the two films can be applied to different areas in the future.

## Figures and Tables

**Table 1.** Molecular weight of the poly(trimethylene carbonate) (PTMC) and poly(lactide trimethylene carbonate) (PLA-TMC) polymers used for the experiments.

Polymer	*M*_n_ [kDa]	*M*_w_ [kDa]	PDI
PTMC	6.7	10.8	1.62
P(LA-TMC)-5	51.6	83.4	1.62
P(LA-TMC)-7	58.6	94.7	1.59
P(LA-TMC)-9	91	143	1.57

**Table 2.** The effects of the temperature and screw speed on the film blowing of ploy(lactide-*co*-trimethylene carbonate).

Stage I (°C)	Stage II (°C)	Stage III (°C)	Stage IV (°C)	Screw speed (pr/min)	Macroscopic morphology of film
100	140	140	130	40	Film with white points
110	150	150	137	40	Smooth film
120	160	160	140	40	Unformed film
110	150	150	137	20	Unformed film
110	150	150	137	60	Porous film

**Table 3.** The effects of the temperature and screw speed on the film blowing of polylactide/polytrimethylene carbonate.

StageI (°C)	Stage II (°C)	Stage III (°C)	Stage IV (°C)	Screw speed (pr/min)	Macroscopic morphology of film
160	175	175	165	40	Film with white points
170	185	185	175	40	Smooth film
180	195	195	185	40	Unformed film
170	185	185	175	20	Unformed film
170	185	185	175	60	Porous film

**Table 4.** The initial decomposition temperature and decomposition temperature of PLA, P(LA-TMC) films and PLA/PTMC films

Polymer	Initial decomposition temperature/°C	Decomposition temperature/°C
PLA	315	364
P(LA-TMC)-9	275	340
P(LA-TMC)-7	265	328
P(LA-TMC)-5	259	310
PLA/PTMC-9	284	339
PLA/PTMC-7	283	329
PLA/PTMC-5	276	325

**Table 5.** Weight Loss, firmness, pH value, color, and sensory scores of apples.

Film		P(LA-TMC)-7	PLA/PTMC-7	LDPE	Control
Weight loss (%)	0	0	0	0	0
	7	1.9 ± 0.4 ^a^	1.8 ± 0.2 ^a^	2.0 ± 0.1 ^a^	5.6 ± 0.4 ^b^
	14	3.0 ± 0.2 ^a^	3.2 ± 0.2 ^a^	2.9 ± 0.0 ^a^	9.2 ± 0.6 ^b^
	21	3.9 ± 0.1 ^a^	3.6 ± 0.1 ^a^	3.6 ± 0.3 ^a^	14.3 ± 0.5 ^b^
	28	7.4 ± 0.3 ^a^	7.2 ± 0.6 ^a^	6.7 ± 0.5 ^a^	20.8 ± 1.3 ^b^
Firmness (N)	0	22.6 ± 0.2 ^a^	22.6 ± 0.2 ^a^	22.6 ± 0.2 ^a^	22.6 ± 0.2 ^a^
	7	21.3 ± 0.1 ^b^	21.9 ± 0.7 ^b^	21.8 ± 1.1 ^b^	19.2 ± 0.3 ^a^
	14	19.4 ± 0.4 ^b^	19.2 ± 0.2 ^b^	19.8 ± 0.9 ^b^	17.3 ± 0.5 ^a^
	21	18.5 ± 0.8 ^b^	18.0 ± 0.8 ^b^	18.7 ± 0.7 ^b^	10.5 ± 0.6 ^a^
	28	16.5 ± 1.0 ^a^	16.1 ± 0.4 ^a^	15.9 ± 0.1 ^a^	--
Acidity	0	1.24 ± 0.03 ^a^	1.24 ± 0.03 ^a^	1.24 ± 0.03 ^a^	1.24 ± 0.03 ^a^
	7	1.21 ± 0.11 ^b^	1.20 ± 0.09 ^b^	1.21 ± 0.02 ^b^	1.16 ± 0.05 ^a^
	14	1.15 ± 0.07 ^b^	1.14 ± 0.07 ^b^	1.14 ± 0.12 ^b^	1.07 ± 0.13 ^a^
	21	1.12 ± 0.08 ^b^	1.11 ± 0.15 ^b^	1.12 ± 0.06 ^b^	1.05 ± 0.09 ^a^
	28	1.10 ± 0.06 ^a^	1.09 ± 0.07 ^a^	1.09 ± 0.07 ^a^	--
Sensory evaluation	0	9.8 ± 0.2 ^a^	9.8 ± 0.2 ^a^	9.8 ± 0.2 ^a^	9.8 ± 0.2 ^a^
	7	8.9 ± 0.4 ^b^	8.8 ± 0.8 ^b^	9.0 ± 0.6 ^b^	7.5 ± 0.2 ^a^
	14	8.1 ± 0.6 ^b^	8.1 ± 0.2 ^b^	7.9 ± 0.7 ^b^	6.1 ± 0.8 ^a^
	21	6.5 ± 0.7 ^b^	6.8 ± 0.4 ^b^	7.0 ± 0.3 ^b^	3.3 ± 0.1 ^a^
	28	5.8 ± 0.1 ^a^	5.9 ± 0.5 ^a^	6.2 ± 0.6 ^a^	--

All data are presented as mean ± standard deviation of the three replicates. Values followed by different small letter (a, b) in the same row are significantly different (*p* < 0.05). -- not detected.
